# Successful resection of a huge brainstem enterogenous cyst: case report and literature review

**DOI:** 10.3389/fonc.2024.1485221

**Published:** 2024-12-20

**Authors:** Boyuan Huang, Yanming Miao, Weifeng Jia, Mengyang Wang, Cong Ren, Wei Zeng, Liangning Shui, Miao Zong

**Affiliations:** Department of Neurosurgery, Beijing Electric Power Hospital, Beijing, China

**Keywords:** enterogenous cysts, brainstem, paediatric, surgery, intracranial

## Abstract

Enterogenous cysts (ECs) are rare, benign, congenital ectopic endodermal cysts that only occasionally involve the central nervous system. We presented the diagnosis and treatment of an exceedingly rare case of EC located in the brainstem, which has previously been reported only seven times in pediatric patients. The patient underwent complete surgical resection and experienced no recurrence during the 6-month follow-up. Histopathological examination showed a characteristic cyst wall lined by a monolayer, pseudocompound cubic or columnar epithelium. Preoperative diagnosis of brainstem EC is still challenging. Surgical resection is an effective treatment, and radiotherapy and chemotherapy have not shown reliable therapeutic efficiency.

## Introduction

Enterogenous cysts (ECs) are rare, benign, congenital ectopic endodermal cysts that are lined by gastrointestinal or respiratory mucin-secreting epithelium. ECs typically occur in the mediastinum and abdomen, and are infrequently found in the central nervous system (CNS) (1). In the CNS, ECs are usually located extramedullary in the spinal cord and rarely located intracranially, which are commonly found in the cerebellopontine cistern, pre-pontine cistern, or ventriculus quartus cerebri (2). ECs occur more often in male adolescents, with a male-to-female ratio of 3:1 to 2:1 (2). Here, we reported an extremely rare case of EC that was located in the brainstem of a 17-year-old male adolescent. Before that, only seven reports have described brainstem ECs in pediatric patients (0–18 years) (**Table 1**). Among those cases, only four patients had achieved tumor total resection, and our case has the largest brainstem EC that had been completely resected.

**Table 1:** Case reports of brainstem ECs.

Case	Author	Time	symptom	Age	MR T1 signal	Intracystic nodule	Extent of Excision	Duration of recurrence
1	Samuel, et al. (3)	2024	headache	10 yr	hyperintense	Yes	Subtotal	NR at 1 year
2	Agresta, et al. (4)	2020	dysphagia, developmental retardation	16 mo	hypointense	No	Subtotal	NR at 9 mo
3	Shimizu, et al. (5)	2019	headache,diplopia,dysarthria	7 yr	hypointense	No	Subtotal	NR at 5 yr
4	Wong, et al. (6)	2016	facioplegia, diplopia,hemiparesis	16 mo	hypointense	Yes	Subtotal	NR at 9 mo
5	Birinyi, et al. (7)	2014	facioplegia, dystaxia,	4 yr	hypointense	Non-applicable	Total	NR at 6 mo
6	Ko, et al. (8)	2008	intermittent headache and vomiting	4 yr	hypointense	Yes	Total	Non-applicable
7	Oliveira, et al. (9)	2005	diplopia	5 yr	Non-applicable	Non-applicable	Total	NR at 1 yr
8	Oliveira, et al. (9)	2005	diplopia	11 yr	Non-applicable	Non-applicable	Total	NR at 4 yr

NR, Non-recurrence; Yr, year; Mo, month.

## History and imaging

**Figure 1:**
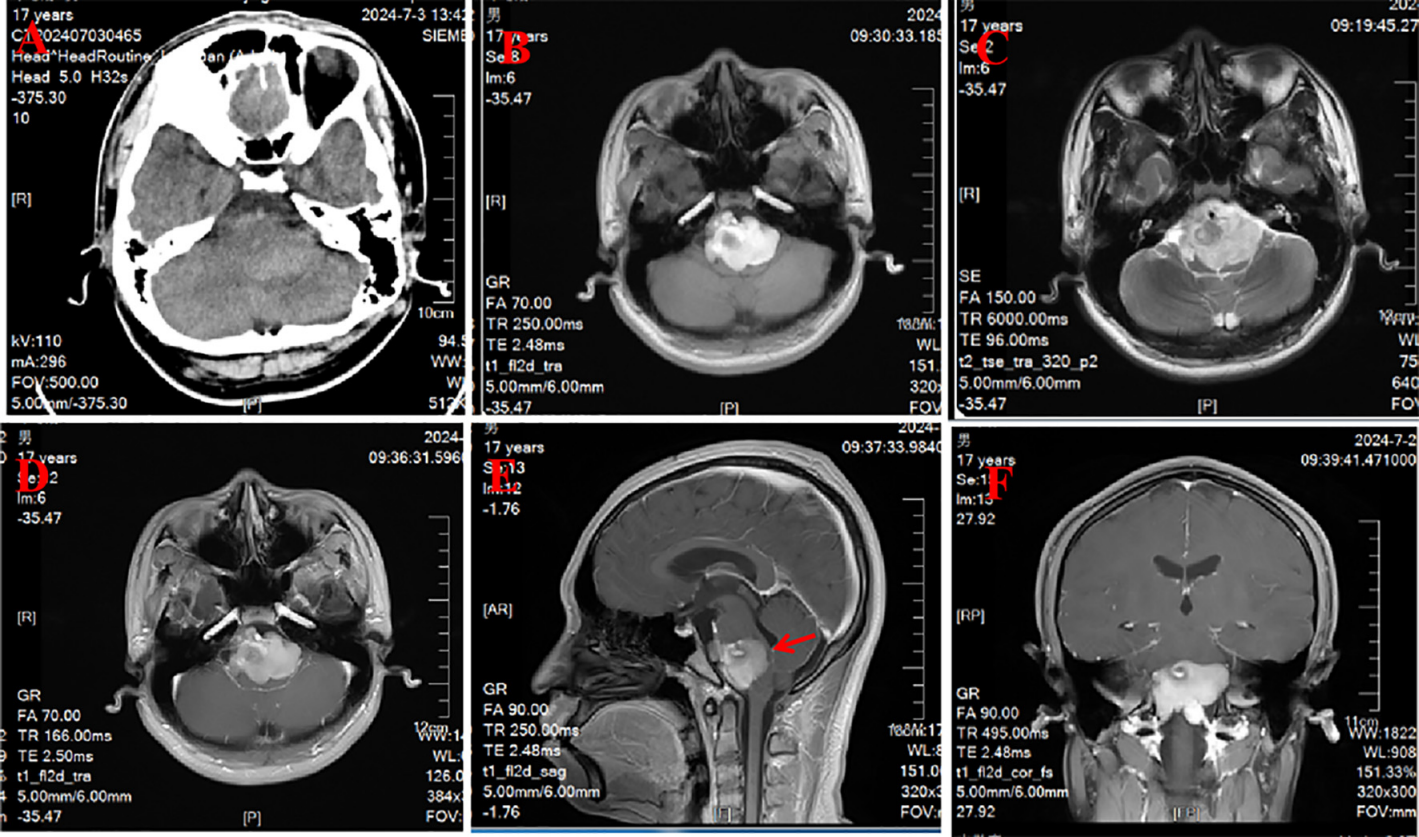
Preoperative CT and MRI results. **(A)** CT scanning showed a cauliflower-like hyperdense lesion located in the brainstem. **(B, C)** The lesion was mostly hyperintense on T1- and T2-weighted scanning. **(D–F)** Enhanced scanning showed that the lesion was a prominent gadolinium enhancement.

A 17-year-old male patient presented in our outpatient with 6 months of dizziness and 12 months of right limb weakness, which was aggravated and resulted in the inability to flex the right lower limb and unsteady walking for 1 month. The patient, without any family history or medical history, was diagnosed by the local hospital as having a brainstem tumor and was transferred to our hospital. The physical and neurological examinations showed that the muscle strength of his limbs was class V on the left side, class IV in the extensors, and class II in the flexors on the right side. Apart from these, no other positive signs were detected in the examinations. Head computed tomography (CT) scanning showed a cauliflower-like hyperdense lesion located in the brainstem (**Figure 1A**). On magnetic resonance imaging (MRI), the lesion was extracerebral and located in the ventral side of the brainstem, compressing the brainstem evidently. The lesion was mostly hyperintense on T1- and T2-weighted scanning, with prominent gadolinium enhancement (**Figures 1B, C**). Moreover, there was a small nodule in the right part of the lesion that showed a low signal on T1, T2, and enhanced scanning, suggesting a calcification within the lesion. The lesion measured approximately 47 × 38 × 36 mm in size, which severely compressed the brainstem, causing it nearly to be a straight line with only 3 mm at the narrowest point (**Figures 1D–F**). Based on the above clinical evidence, the patient was preoperatively diagnosed as having an epidermoid cyst. Because of the evident clinical symptoms and prominent mass effect on the brainstem, we decided to operate on this patient.

## Surgical details and histopathology

The patient was positioned laterally and surgery was performed using a far lateral approach, craniotomy with an extension from the occipital midline through the foramen magnum to the left condyle of occipital bone, ending in the star point covering the transverse sinus. After partially removing the occipital condyles, incising the posterior arch of the atlanto-axial, and cutting the endocranium, we explored the left ventral side of the cerebellum using a microscope. The lesion was exposed in the ventral part of the pons, which was embedded in the pons and severely compressed, displaced, and deformed it. The mass was cystic, with an intact outer envelope, and a thick yellowish cystic liquid content. Fortunately, the cyst wall did not adhere tightly to the brainstem. Therefore, after aspirating the cyst fluid, we carefully stripped the cyst wall from the brainstem and removed the cyst completely.

**Figure 2:**
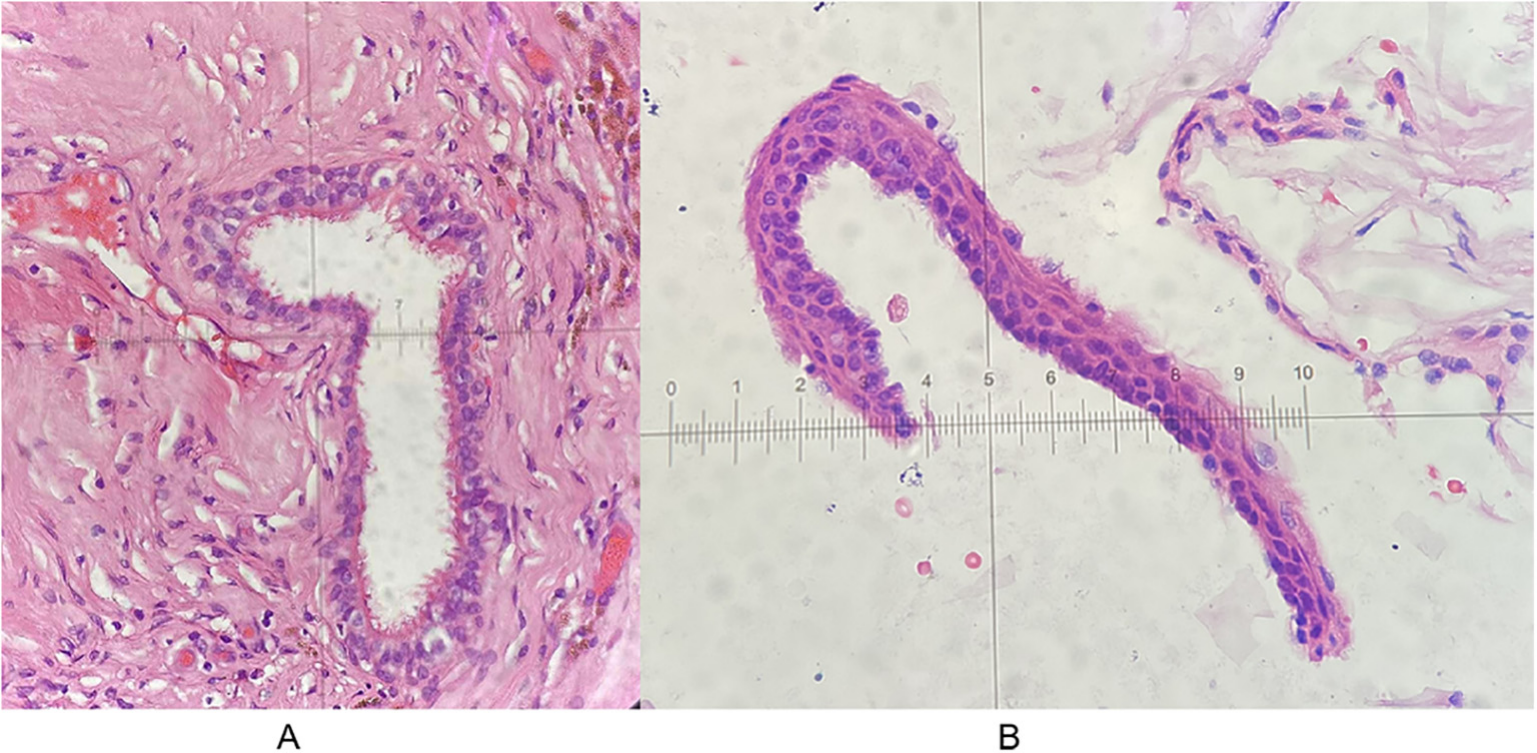
Histological results of the lesion. **(A)** 10 × 20. **(B)** 10 × 40.

**Figure 3:**
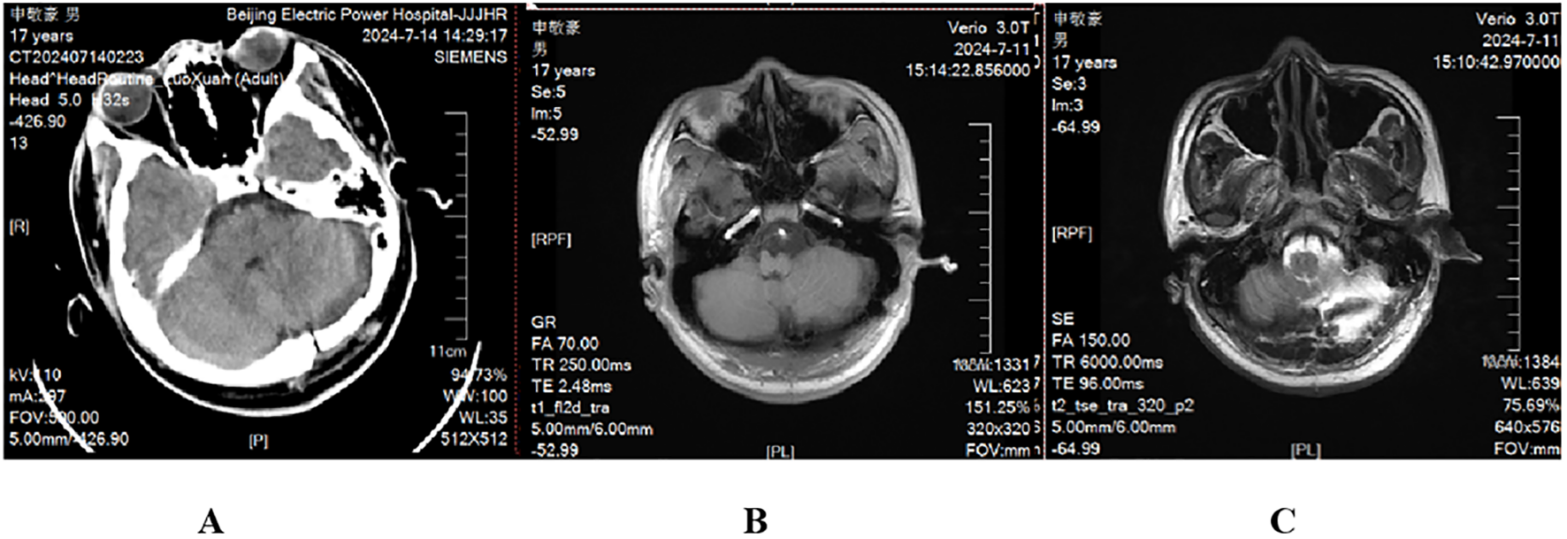
Postoperative CT and MRI results. CT scanning **(A)** and T1-weighted **(B)** and T2-weighted **(C)** scanning showed that the lesion was completely resected.

Microscopically, the cyst wall was covered with pseudo-stratified epithelium with abundant foam cells around. Under the epithelium is fibrous tissue, with collagen, as well as little capillaries between tissues (**Figure 2**). In the cyst fluid, there were a large number of erythrocytes and a small number of leukocytes. Postoperatively, CT and MRI showed that the cyst was completely removed (**Figure 3**). After the postoperative treatment including lumbar puncture, postoperative limb function exercise, and neurotrophic drug administration, the patient’s symptoms have partially improved with a muscle strength of class IV in the flexors on the right side, compared with that of class II before the surgery. The patient and his parents were very satisfied with the treatment. There was no tumor progression over the next 6 months of follow-up, nor were there any recurrences. When he came back 6 months later, his symptoms have totally improved with a muscle strength of class V on the limbs.

## Discussion

ECs are defined as benign cystic lesions lined by gastrointestinal or respiratory mucin-secreting epithelium, which were first found by Puussepp in 1934, but were officially named enterogenous cysts in 1958 by Harriman (10, 11). The pathogenesis of ECs is still controversial, which most researchers believed should be ascribed to the mutation or hypoplasia of the ectoblast and entoderm during the third embryonic week (2). From the beginning of the third embryonic week, the ectoblast, gradually developing with neural tubes, and the entoderm, differentiating into the intestinal tube, will divide from the ectoblast along with the embryonic development. In the case of impaired, vestigial, or ectopic embryo development, EC will form, which is usually combined with gastrointestinal, spinal, or medullispinal malformations (2). ECs can occur at any age, with more prevalence in male patients (2, 12). However, ECs of male patients are more likely to be found in the spine, and those of female patients are more likely to be found intracranially (12). ECs of CNS mostly occur in the spinal canal (more than 80%), accounting for 0.3% to 0.5% of the intraspinal tumor, with a higher prevalence in the cervical and upper thoracic segments, which preferably occur in the ventral part of the subdural spinal cord (12). In our case, the patient was a male adolescent, who was found to have an EC in a very rare location of the brainstem without any other intracranial malformations. Until now, there have been only seven cases of brainstem ECs reported in pediatric patients. Among these cases, only four had achieved total tumor resection, and the tumor in our case is the largest in size among the completely resected tumors.

The clinical manifestations of ECs are closely related to the pathogenic site, with long-term recurrent headache being the first symptom in most cases. Later on, along with cyst enlargement, the mass effects gradually appear, causing epilepsy, intracranial hypertension, and dysneuria. Even more, aseptic meningitis may occur in case of a fistula formation.

The diagnosis of intracranial ECs relies mainly on imaging and pathology. Because of the slow growth of cysts, low incidence, variable clinical symptoms, and atypical imaging manifestations, it is difficult to distinguish them from other intracranial mass lesions, which leads to a difficult preoperative diagnosis. CT scanning can only manifest the location of the lesion and the feature of cystic changes. MRI scanning can distinctly show the shape of the lesion and its relationship to the surrounding brain tissue. On MRI scanning, most ECs of CNS have thin, uniform walls, and smooth margins, with little edema in the surrounding brain tissue. On T1- and T2-weighted scanning, the cyst usually displays a signal equal to or slightly higher than that of the cerebrospinal fluid (CSF), with no enhancement or slight enhancement in case of fibrillation of partial cyst wall on enhanced scanning (10, 13). In our case, the lesion displayed marked hyperintensity on both T1- and T2-weighed scanning with prominent gadolinium enhancement, which is not in accordance with the reported cases. This nonconformity of signals in MRI may be attributed to the high protein content in cystic fluid.

Histologically, ECs can be classified into three groups based on the histological origin of cyst epithelium. Group I: The cyst walls are lined by a monolayer, pseudocompound cubic or columnar epithelium, with or without fiber hairs. Group II: The cysts, in addition to the above structure, may be composed of mucous glands, plasma glands, and ganglia. Group III: The cysts, in addition to the findings in group II, may be composed of ventricular membrane and neuroglial components (14). In immunohistochemical staining, EC cells show positive expression of epithelial membrane antigen and carcinoembryonic antigen, with negative expression of glial fibrillary acidic protein (GFAP) (15).

As a congenital disease, ECs should be distinguished from intracranial dermoid cysts, epidermoid cysts, and arachnoid cysts. Dermoid cysts are usually seen in adults (at least 40 years old), with the sellar region or cranial fossa being the common location (16). On account of their fatty component, dermoid cysts display heterogeneous signals on MRI scanning, which often shows a slight hypointense signal or an occasionally hyperintense signal on T1-weighted scanning, and a slight hyperintense signal on T2-weighted scanning, without gadolinium enhancement. The imaging displays of epidermoid cysts are multifarious, depending on the composition and proportion of the cysts, which usually show a hypointense signal (slightly higher than the CSF signal) on T1-weighted scanning and a hyperintense signal on T2-weighted scanning (17). The arachnoid cysts display signals similar to the CSF, which are hypointense signals on T1-weighted and hyperintense signals on T2-weighted scanning. In addition, the expression of GFAP can be used for differential diagnosis from ECs and arachnoid cysts.

The treatment of ECs is based on surgical resection, with the goal of complete removal of the lesion. Even if the cyst is completely removed, it may still recur, worsening the symptoms of neurological deficit and increasing the risk and difficulty of reoperation (18–20). Here, we present several operative experiences: (1) To avoid aseptic inflammation, tampons should be applied around the cyst during excision to prevent leakage of cystic fluid, and the operative region should also be repeatedly irrigated after the resection. (2) During the operation, we found that the cyst has severely compressed the brainstem, significantly deforming it. However, the cyst wall was not tightly adherent to the brainstem, which resulted in the complete excision of the cyst. Nevertheless, we still do not recommend total resection of brainstem ECs at the expense of damage to the surrounding tissues, especially the brainstem. With regard to the cyst being tightly adherent to the brainstem, we suggest that partial resection should be performed first, followed by electrocautery of the remaining part of the cyst wall. (3) Although ECs are considered as benign lesions, there have already been reports about cases with recurrence, dissemination, and canceration (18, 19). Therefore, intraoperative freezing pathological examination is proposed, which plays an important role in the selection of the operative approach, the resection scope, and the prognosis of intracranial ECs.

## Conclusion

We present a complete surgical resection of a rare huge brainstem EC, in which total resection had been reported in only four cases. Intracranial ECs are congenital benign lesions, the diagnosis of which fundamentally relies on imaging and histological results. Signals of ECs on MRI are multiple, depending on the content of the cyst. Typically, the pathological characteristic of ECs is the presence of cyst walls lined by a monolayer, pseudocompound cubic or columnar epithelium, which can be further classified into three groups. Surgical resection is still the primary treatment for intracranial ECs, since radiotherapy and chemotherapy have not been proven to have a therapeutic effect. Therefore, further investigations are needed to optimize management and recurrence avoidance.

## Data Availability

The original contributions presented in the study are included in the article/**Supplementary Material**. Further inquiries can be directed to the corresponding author.
