# Supplementary material for: Successful resection of a huge brainstem enterogenous cyst: case report and literature review
Source: Front Oncol. 2024 Dec 20;14:1485221. doi: 10.3389/fonc.2024.1485221 (PMC11696278; doi:10.3389/fonc.2024.1485221)
Supplement: Supplementary file 1 [file Presentation1.pptx]

## Slide 1
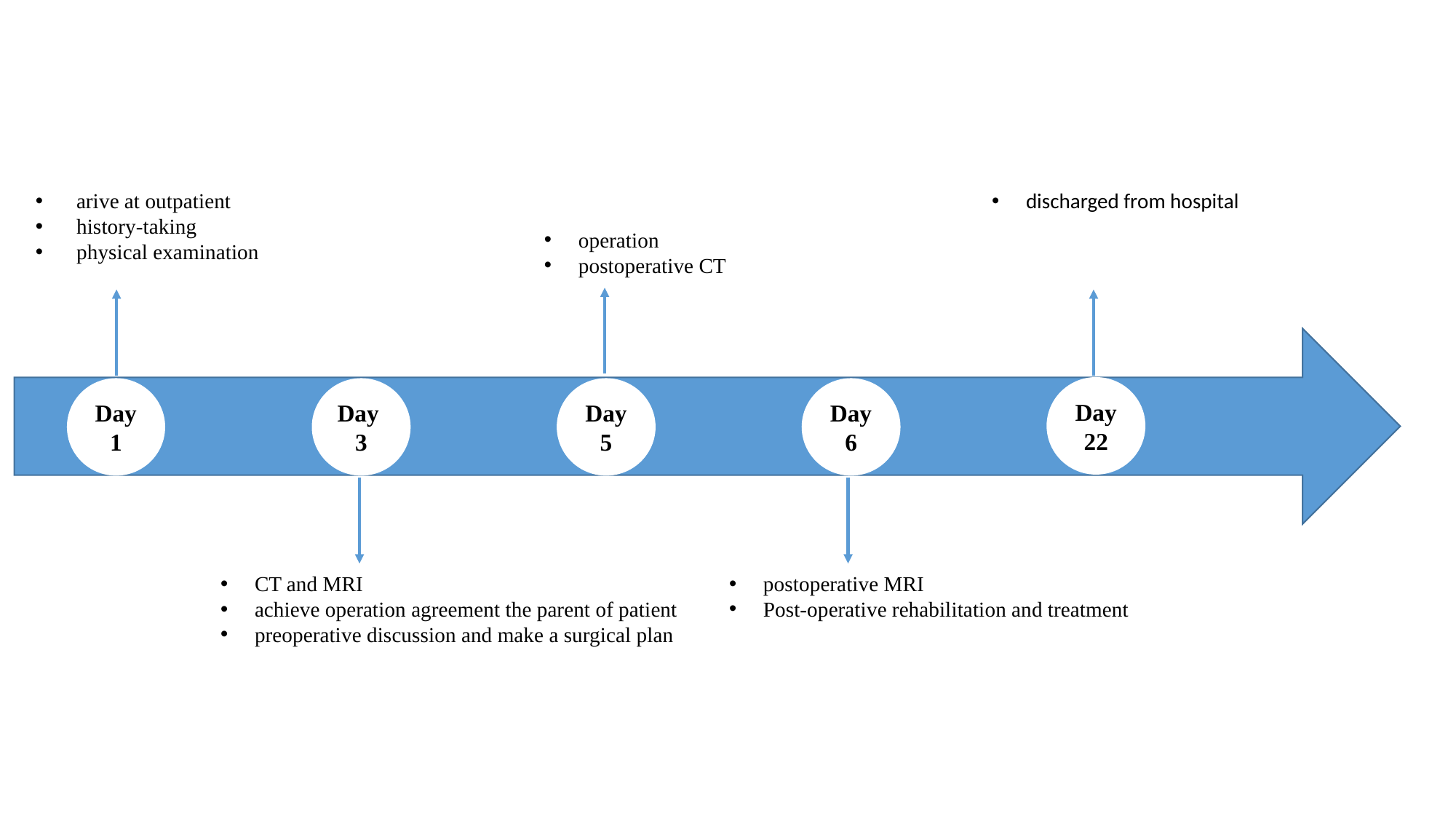

arive at outpatient
history-taking
physical examination
discharged from hospital
operation
postoperative CT
Day 22
Day 1
Day
3
Day 5
Day 6
postoperative MRI
Post-operative rehabilitation and treatment
CT and MRI
achieve operation agreement the parent of patient
preoperative discussion and make a surgical plan
